# An Act of Humanity

**Published:** 1875-06

**Authors:** 


### AN ACT OF HUMANITY.

Those of our readers who have perused our Magazine for a year past, have learned our views concerning a certain medical preparation which has been somewhat conspicuously advertised in our pages, under the name of "The Van Deren Remedy." They know that we esteem the "Milky Granules" as a safe and simple preparation for all bowel disorders of children, and that we believe few deaths need occur from this class of diseases, if these pellets were in general use.

It is no doubt because of our knowledge of the composition of this "Remedy," and our thorough belief in its efficacy, that its proprietors, Messrs. Hall & Ruckle, the well-known Importers of Choice Medicines, whose great house is at Nos. 218 and 220 Greenwich Street, in this city, have called upon us with a proposition, which we will submit to our readers.

They invite us to distribute for them, free, 5,000 parcels of this Remedy, to parents having infants or young children. They supply the medicine, in small packages, with printed instructions, and ask us to hand them gratuitiously to all who apply. They also request to us to prepay and send the packages to such as apply by mail, provided a 3-cent stamp accompanies the application.

We have thought the matter over, and have decided to accept the trust, and thus become the almoners of this bounty.

We shall therefore give to each caller who desires it, a package of these small granules. We shall mail a like package to all who apply by letter, with the 3-cent stamp enclosed for the return postage.

Only a single condition attends this distribution, and that is, that all who administer this Remedy, shall, with little delay, write us the result, giving the name, age, and address of the little patient. These letters we will make the basis of an article in our journal at some future day.

We imagine that a great deal of real good will be accomplished by this distribution. If it is accepted and availed of in the spirit in which it is proposed, the lives of a good many children will be saved this summer, which would otherwise be lost. We are well acquainted with the "Remedy," and know it to be simple and safe. We have employed it constantly, in Cholera-Infantum and the severe bowel complaint of children, and it has never disappointed us. We have at hand a large pile of letters from parents and physicians, all bearing testimony to the value of the "Remedy."

With all this evidence before us, we feel that we should be culpably negligent if we declined the trust imposed, or hesitated to further an undertaking so well calculated to lessen suffering and save the lives of innocent children, during the trying summer months now at hand.

